# The impact of Covid-19 in patients with chronic myeloid leukemia—a nationwide population-based study

**DOI:** 10.1038/s41375-023-01893-1

**Published:** 2023-04-10

**Authors:** Torsten Dahlén, Hjalmar Flygt, Anna Lübking, Ulla Olsson-Strömberg, Lovisa Wennström, Arta Dreimane, Anders Själander, Susannah Leach, Magnus Gisslén, Huiqi Li, Martin Höglund, Leif Stenke, Fredrik Nyberg

**Affiliations:** 1 Department of Medicine Solna, Clinical Epidemiology Division, Karolinska Institutet, Stockholm, Sweden; 2 Department of Hematology, Karolinska University Hospital, Stockholm, Sweden; 3 Department of Medical Science and Division of Hematology, University Hospital, Uppsala, Sweden; 4 Department of Hematology, Oncology and Radiation Physics, Skåne University Hospital, Lund, Sweden; 5 Section of Hematology, Sahlgrenska University Hospital, Gothenburg, Sweden; 6 Department of Hematology, Linköping University Hospital, Linköping, Sweden; 7 Department of Public Health and Clinical Medicine, Umeå University, Sundsvall, Sweden; 8 Department of Clinical Pharmacology, Sahlgrenska University Hospital, Gothenburg, Sweden; 9 Department of Microbiology and Immunology, Institute of Biomedicine at Sahlgrenska Academy, University of Gothenburg, Gothenburg, Sweden; 10 Department of Infectious Diseases, Institute of Biomedicine, Sahlgrenska Academy, University of Gothenburg, Gothenburg, Sweden; 11 Department of Infectious Diseases, Region Västra Götaland, Sahlgrenska University Hospital, Gothenburg, Sweden; 12 School of Public health and Community Medicine, Institute of Medicine, Sahlgrenska Academy, University of Gothenburg, Gothenburg, Sweden

**Keywords:** Epidemiology, Prognosis

## To the Editor:

Since December 2019 more than 460 million confirmed cases and 6 million deaths have been attributed world-wide to *coronavirus disease 2019* (Covid-19) [1]. Patients with hematological cancer have been reported to suffer increased risk of severe Covid-19, with a hospitalization rate of more than 50% and a case fatality rate of approximately 30% [2]. The impact of Covid-19 on patients with chronic myeloid leukemia (CML) remains uncertain [2–5]. The Italian Campus CML network reported on more than 8000 CML patients and detected only 217 Covid-19 positive cases [5]. However, among these cases 22% were admitted to hospital with a relatively high mortality rate of 5.5%. Similarly, a preliminary report from the CANDID study, conducted by the International CML Foundation, estimated the mortality rate among Covid-19 infected CML cases at 14% [4]. Because of these somewhat diverging data, a recent review concluded that the incidence and severity of Covid-19 in CML have not yet been thoroughly investigated and called for a true population-based study [6]. Our goal was to perform such a study, examining a large number of patients and comparing against carefully matched controls, utilizing high-quality full-coverage population-based registers addressing the period when Covid-19 was classified as “disease dangerous to public health” with widespread public PCR testing was available, February 1st 2020 to April 1st 2022.

We utilized an existing database containing data on all Swedish individuals together with information on relevant Covid-19 outcomes (the SCIFI-PEARL project database), with information on all positive Covid-19 tests (PCR-based testing) as well as all Covid-19-related hospital and intensive care unit (ICU) admissions, deaths, and national vaccination data [7]. This database specifically contains exposure and outcome information in terms of information on specialized outpatient- and all inpatient health-care, cancer diagnoses, as well as information on all dispensed drug prescriptions [8].

We defined two separate CML cohorts. The first, main cohort, consisted of individuals diagnosed with TKI-treated CML in chronic phase (CP-CML) before March 1st 2020, defined here as the start of the pandemic in Sweden. A secondary cohort consisted of individuals diagnosed during the pandemic, after this index date. We excluded individuals born outside of Europe, as it is documented that they suffered increased susceptibility to severe Covid-19 outcomes in the Swedish population [9]. Using the same database, we selected five controls for each CML patient in the two cohorts by randomly matching for age (within 60 days), sex, and county of residence on March 1st 2020. The individuals in the two CML and control cohorts were followed from March 1st 2020 to the first event of each outcome studied, April 1st 2022, emigration or death, whichever occurred first. This effectively means that follow-up for patients diagnosed during the pandemic was also initiated at the start of the pandemic and not at the time of the CML diagnosis – allowing to capture potentially more severe Covid-19 cases even if diagnosed before the CML disease – a disease that is diagnosed in the asymptomatic case in half of the patients and that has existed and affected the body for years before the diagnosis date.

We performed multiple sets of analyses, comparing the incidence rate ratios (IRRs) between the CML and control cohorts. In the first main analysis, elucidating the inherent risk of Covid-19 in an unvaccinated CML population, patients were additionally censored at the time of first vaccination. Four separate outcomes were investigated: the risk of testing positive for Covid-19, hospital or ICU admission, respectively, related to Covid-19 (defined as hospital or ICU admission with Covid-19 as a primary or secondary diagnosis within 60 days of the first positive Covid-19-test) or death (defined as death with Covid-19 as underlying or contributing cause of death during follow-up).

In several secondary analyses, we investigated the same four outcomes. First by initiating follow-up at the time of vaccine protection (defined as 14 days after the 2nd dose of any vaccine used), comparing the CML cohort to their control cohort. To reduce the impact of the knowledge gap regarding the management of Covid-19 disease early in the pandemic and the potential for less adequate management, we also performed analyses limiting the population to CML patients and their respective controls who were alive and without any of the outcomes after the first 6 months of the pandemic. We also compared the cohort consisting of patients diagnosed during the pandemic to their matched control cohort to elucidate the impact of a more active, recent leukemic disease. Furthermore, we investigated the effect of the first-line TKI imatinib as compared to the later generations TKIs (dasatinib, nilotinib, bosutinib, or ponatinib) at the time of Covid-19-infection, limiting the analysis to the CML population. IRRs were calculated using a quasi-Poisson regression model incorporating sex (categorical) and age (as a restricted cubic spline with 3 equally placed knots), in the main analysis to model the events comparing the CML cohort with the matched control cohort. In the analysis within the CML cohort, the model incorporated the same covariates with the addition of TKI treatment as a categorical time-dependent variable (imatinib or any other TKI). A total of 1146 CP-CML patients were identified and 5,730 matched controls were selected. Among them, 83% of the CML patients were diagnosed before the index date March 1st 2020. The characteristics of patients and controls are outlined in Table 1. The median follow-up from the index date was 1.3 years for all groups.

**Table 1:** Characteristics of Chronic Myeloid Leukemia (CML) patients and matched population controls in Sweden, at the start of the Covid-19 pandemic (1 March 2020).

	CML cohorts		Matched cohorts	
	Diagnosed before the pandemic	Diagnosed during the pandemic	Controls for patients diagnosed before pandemic	Controls for patients diagnosed during the pandemic
**Total,** ***N***	948	198	4740	990
**Female,** ***N*** **(%)**	430 (45%)	94 (47%)	2150 (45%)	470 (47%)
**Age years, median (IQR)**	64 (51–74)	63 (51–72)	64 (51–74)	63 (51–72)
**Age,** ***N***				
<40 years	92 (10%)	26 (13%)	460 (10%)	130 (13%)
40–65 years	420 (44%)	84 (42%)	2100 (44%)	420 (42%)
65–75 years	240 (25%)	55 (28%)	1200 (25%)	275 (28%)
>75 years	196 (21%)	33 (17%)	980 (21%)	165 (17%)
**Vaccinated,** ***N*** **(%)**	894 (94%)	180 (91%)	4367 (92%)	893 (90%)
**Follow-up years, median (IQR)**	1.3 (1.3–1.4)	1.3 (1.3–1.4)	1.3 (1.3–1.4)	1.3 (1.3–1.5)
**Diagnosis date,** ***N***				
<2007	223 (24%)			
2008–2012	215 (23%)			
2013–March 1st, 2020	510 (54%)			
After March 1st 2020		198 (100%)		
**TKI at start of pandemic,** ***N*** **(%)**				
Imatinib	548 (58%)			
≥2nd generation TKI^a^	400 (42%)			

*IQR* interquartile range, *TKI* tyrosine kinase inhibitor.

^a^2nd generation TKI included dasatinib, nilotinib, bosutinib, and ponatinib.

The risk of Covid-19 infection and severe Covid-19 disease among CML patients is summarized in Table 2. The risk of testing positive for Covid-19 was not significantly increased in CML patients. Among unvaccinated CML patients, 106 (11.1%) developed positivity, as compared to 448 (9.5%) among controls. However, CML patients in the main cohort (i.e., diagnosed prior to the index date) and with no Covid-19-vaccination showed an increased risk of hospitalization for Covid-19, as compared to controls; IRR 1.93 (95% confidence interval [CI], 1.17–3.18, *P* = 0.01). Among the 24 unvaccinated CML patients admitted to the hospital due to Covid-19, all had comorbid conditions. The most common comorbid conditions were cardiovascular diseases (*N* = 15), renal disease (*N* = 12), diabetes (*N* = 10), and chronic lung disease (*N* = 8). A similar point estimate, but due to lower statistical power, non-significant trend was observed for mortality with IRRs for Covid-19-related death 2.04 (95% CI, 0.91–4.56). This corresponds to a case fatality-rate of 1.9% (*N* = 2) and 1.1% (*N* = 5) for CML patients and controls, respectively. None of the CML patients who tested Covid-19 positive were admitted to an ICU.

**Table 2:** Risk of Covid-19 infection and severe Covid-19 disease in patients with chronic myeloid leukemia (CML) in Sweden during the period March 1st 2020 to April 1st 2022.

	Risk of testing positive for Covid-19			Risk of hospital admission			Risk of ICU admission			Risk of Covid-19 death		
Diagnosed before pandemic/before index date	Events, cases	Events, controls	IRR (95% CI)	Events, cases	Events, controls	IRR (95% CI)	Events, cases	Events, controls	IRR (95% CI)	Events, cases	Events, controls	IRR (95% CI)
	*N*	*N*		*N*	*N*		*N*	*N*		*N*	*N*	
**Unvaccinated**	106	448	1.23 (0.82–1.83)	24	64	1.93 (1.17–3.18)*	0	10	–	2	5	2.04 (0.91–4.56)
**Vaccinated**	36	195	0.88 (0.60–1.29)	4	9	2.22 (0.66–7.47)	0	0	–	0	0	–
**6 months delayed-entry**	76	430	0.89 (0.54–1.48)	11	59	0.95 (0.45–2.01)	0	9	–	2	5	2.03 (0.92–4.49)
**Diagnosed during pandemic/after index date**	10	89	0.55 (0.28–1.1)	3	15	0.99 (0.33–2.99)	0	3	–	0	1	–

	Events,imatinib	Events,> 2 G TKI	IRR (95% CI)	Events,imatinib	Events,> 2G TKI	IRR (95% CI)	Events,imatinib	Events,> 2G TKI	IRR (95% CI)	Events,imatinib	Events,>2G TKI	IRR (95% CI)
**Imatinib vs.** ≥ 2G TKI*	51	55	1.22 (0.73–2.05)	13	11	1.09 (0.4–2.96)	0	0	–	0	2	–

Risks are for CML patients vs. matched population controls, and among CML patients for 1st (imatinib) vs. ≥ 2nd generation TKI:s*.

The 6-month delayed-entry model consisted of CML patients and their controls where none of the patients or controls developed Covid-19 in the first 6 months of the pandemic. *IRR i*ncidence rate ratio, *CI* confidence interval. *P = 0.01.

*ICU* intensive care unit.

*2G TKI included dasatinib, nilotinib, bosutinib and ponatinib.

In the analysis where we delayed entry with 6 months, to account for incomplete testing and a high mortality rate early in the pandemic phase, the effect of increased Covid-19 hospital admission disappeared.

The vaccination rate during the follow-up was high among both CML patients and controls; 94% and 92%, respectively. Initiating follow-up at the time of vaccination for each subject, there was no significant difference between the CML and the control cohorts regarding any of the outcomes, likely due partly to fewer events, although point estimates were consistent with the ones for unvaccinated individuals.

Focusing on the 198 CML patients diagnosed after the index date March 1st 2020 (i.e., during the pandemic) we identified no increased risk vs. their 948 matched controls for any of the listed outcomes Studying the effect of imatinib vs. later generation TKIs we detected no significant differences related to any of the Covid-19 outcomes. In a sensitivity analyses relaxing the definition of hospital admission and/or ICU admission to not require a previous positive PCR test, we did not see any altered findings.

In summary, in this register-based matched cohort study utilizing high-quality nationwide data from a large number of individuals, we investigated the risk of developing Covid-19 and suffering more severe Covid-19 outcomes between CML patients and carefully matched population controls. The overall risks for developing Covid-19 positivity, ICU admission, and case-related mortality were comparable and relatively low. However, in CML patients with no Covid-19 vaccination, we observed an increased risk of hospitalization, as compared to controls. In this study, however, we cannot accurately describe the association between CML and Covid-19 death due to the low number of deaths that occurred. Still, the few deaths observed suggest that if there is an increased risk of death, this increased risk is moderate.

We did not observe any distinct differences regarding Covid-19 outcome comparing CML patients on 1st generation TKI (imatinib) vs. later generations TKI. Moreover, patients diagnosed with CML after the onset of the Covid-19 pandemic (i.e., after the index date March 1st 2020), and thus with a higher leukemic burden, did not display any increased risk of suffering more severe outcomes.

## Data Availability

Due to laws guarding the integrity of Swedish citizens, no patient-level data can be made available. Upon request, aggregate data with different levels of resolution may be possible to provide.
